# Child Mortality Rate in Iran Compared with Other Eastern Mediterranean Countries Based on WHO Report in 2017

**Published:** 2020-01

**Authors:** Ensiyeh JENABI, Salman KHAZAEI

**Affiliations:** 1 Autism Spectrum Disorders Research Center, Hamadan University of Medical Sciences, Hamadan, Iran; 2 Research Center for Health Sciences, Hamadan University of Medical Sciences, Hamadan, Iran

## Dear Editor-in-Chief

The world has reached substantial progress in reducing child mortality in recent decades (1). Every year, preventable causes such as pneumonia, diarrhea and malaria are responsible for millions of death of children under 5 yr of age. More than half of the deaths are associated with malnutrition, while some environmental factors like unsafe water, sanitation and hygiene have significant effect on child mortality indices (2). Child mortality is considered as a key indicator not only for child health but for monitoring overall progress towards the Sustainable Development Goals (SDGs) (3). Therefore, we discuss the status of this index in Iran compared to other Middle Eastern countries according to reported data to the WHO in 2016 (4).

**Fig. 1:**
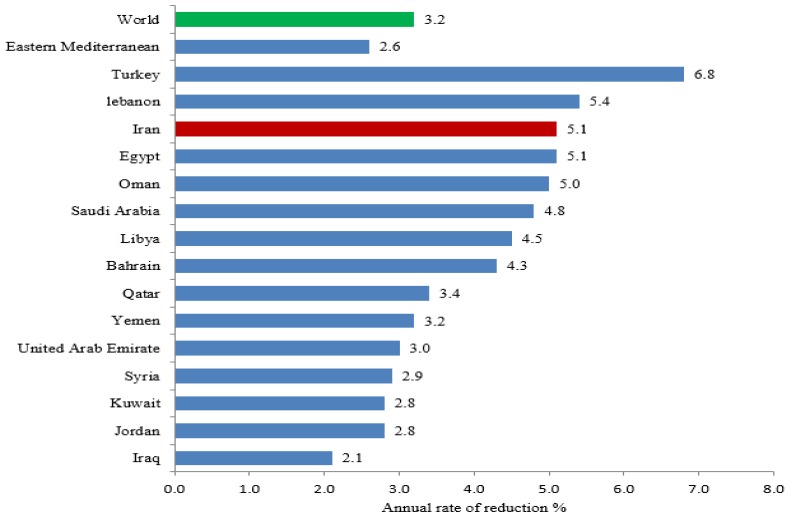
Annual rate of reduction (%) of Under-five mortality rate since 1990–2016 in eastern Mediterranean countries

Globally, the under-five mortality rate dropped from 93 (92, 95) in 1990 to 41 (39–44) deaths per 1,000 live births in 2016, with the 3.2% annual reduction rate. Among countries in this region, United Arab Emirate, Lebanon, Kuwait, Bahrain and Qatar with the 8, 8, 8, 8 and 9 under-five mortality rate respectively per 1000 live births has the better situation and the worst situation belongs to Yemen with the rate of 55 (40–76) (Table 1). While the Turkey, Iran and Egypt with 6.8%, 5.1% and 5.1% have the steepest annual reduction rate from 1990 to 2016, respectively. The lowest decline is related to Iraq (2.1%) during this period has been involved in several civilian and foreign wars (Fig. 1). Except for Lebanon, in the rest of the countries in this region, the percentage of deaths is slightly higher for boys than girls (Table 1). Regarding the infant and neonatal mortality rate per 1000 live births Iran reached 13 and 10 respectively, while United Arab Emirates, Lebanon, Oman, Kuwait and Qatar have managed to reduce both indices to below 10. For both these indices, the worst situation belongs to Yemen with the 43 and 27 infant and neonatal mortality rate per 1000 live births, respectively (Table 1).

**Table 1:** UFMR, IMR and NMR statistics for eastern Mediterranean countries

***Country***	***UFMR(per 1,000 live births) in 2016***	***ARR (percent) 1990–2016 for***	***Sex-specific UFMR (per 1,000 live births)***			***IMR (per 1,000 live births)***	***NMR (per 1,000 live births)***
		**UFMR**	**Male**	**Female**	**Male/Female ratio**		
Egypt	23 (18–29)	5.1	24	22	1.1	19	13
Iran	15 (11–21)	5.1	16	15	1.1	13	10
Turkey	13 (12–13)	6.8	13	12	1.1	11	7
Iraq	31 (23–42)	2.1	34	28	1.2	26	18
Saudi Arabia	13 (7–25)	4.8	14	12	1.2	11	7
Yemen	55 (40–76)	3.2	59	51	1.2	43	27
Syria	18 (14–25)	2.9	19	16	1.2	14	9
Jordan	18 (13–24)	2.8	19	17	1.1	15	11
United Arab Emirate	8 (7–8)	3	9	7	1.3	7	4
Libya	13 (9–19)	4.5	14	12	1.2	11	7
Lebanon	8 (4–14)	5.4	8	8	1.0	7	5
Oman	11 (10–11)	5.0	12	10	1.2	9	5
Kuwait	8 (8–9)	2.8	9	8	1.1	7	4
Qatar	9 (8–9)	3.4	9	8	1.1	7	4
Bahrain	8 (7–9)	4.3	8	7	1.1	20	15
Eastern Mediterranean	52 (46–61)	2.6	55	49	1.1	41	28
World	41 (39–44)	3.2	43	39	1.1	31	19

ARR: Annual rate of reduction, UFMR: Under-five mortality rate, IMR: Infant mortality rate, NMR: Neonatal mortality rate

Disparities in child survival exist across countries in Eastern Mediterranean countries and the countries involved in the war, such as Iraq and Yemen, are not in a good position. Although Iran reached the goals of Millennium Development Goals (MDGs) and the substantial decline occurred for under-five mortality rate, however, compared to some countries in this region require more effort is to further improve these indicators.
